# Evolving Economics: The Erosion of Medicare Reimbursement in Breast Surgery (2003–2023)

**DOI:** 10.1245/s10434-024-15709-8

**Published:** 2024-07-20

**Authors:** Terry P. Gao, Kristen M. HoSang, Richard J. Bleicher, Lindsay E. Kuo, Austin D. Williams

**Affiliations:** 1 Department of General Surgery, Temple University Hospital, Philadelphia, PA 19140 USA; 2 Department of Surgical Oncology, Fox Chase Cancer Center, 333 Cottman Ave., Philadelphia, PA 19111 USA

**Keywords:** Medicare reimbursement, Breast surgery, Breast cancer, Inflation-adjusted, Healthcare economics, Surgical reimbursement

## Abstract

**Introduction:**

Medicare significantly influences reimbursement rates, setting a standard that impacts private insurance policies. Despite declining rates in various specialties, the magnitude of these trends has not been examined in breast surgery. This study examines Medicare reimbursement trends for breast surgery operations.

**Methods:**

Data for 10 breast operations from 2003 to 2023 were collected from the Medicare Physician Fee Look-Up Tool and yearly reimbursement was computed using the conversion factor. The year-to-year percentage change in reimbursement was calculated, and the overall median change was compared with the consumer price index (CPI) for inflation evaluation. All data were adjusted to 2023 United States dollars. The compound annual growth rate (CAGR) was calculated using inflation-adjusted data.

**Results:**

Over the study period, reimbursement for the 10 breast operations had a mean unadjusted percentage increase of + 25.17%, while the CPI increased by 69.15% (*p *< 0.001). However, after adjustment, overall reimbursement decreased by − 20.70%. Only two operations (lumpectomy and simple mastectomy) saw increased inflation-adjusted Medicare reimbursement (+ 0.37% and + 3.58%, respectively). The CAGR was − 1.54% overall but remained positive for the same two operations (+ 0.02% and + 0.18%, respectively). Based on these findings, breast surgeons were estimated to be reimbursed $107,605,444 less in 2023 than if rates had kept pace with inflation over the past decade.

**Conclusion:**

Inflation-adjusted Medicare reimbursement rates for breast surgeries have declined from 2003 to 2023. This downward trend may strain resources, potentially leading to compromises in care quality. Surgeons, administrators, and policymakers must take proactive measures to address these issues and ensure the ongoing accessibility and quality of breast surgery.

Breast cancer continues to be a major public health concern. It is the most common cancer among women and the second leading cause of cancer deaths.^1^ The economic costs associated with breast cancer treatment are equally staggering, placing a strain on both patients and healthcare systems.^2^ Medicare plays a vital role in financing breast cancer treatment for a large segment of this patient population.^3^ Thus, ensuring adequate reimbursement for breast cancer operations is vital to maintaining affordability and quality in breast cancer care.

Past Medicare adjustments, encompassing coverage guidelines, coding practices, and reimbursements, have demonstrably impacted healthcare delivery. Notably, decreasing Medicare reimbursement rates have been observed in numerous surgical subspecialties.^4–8^ These changes may have unintended consequences for patients, such as delayed access to treatments, as well as providers.^9–11^ Moreover, lower reimbursements may result in higher out-of-pocket costs for patients, which creates additional financial barriers for Medicare beneficiaries.^12,13^ Despite the rising prevalence of breast disease and the increasing demand for surgical interventions, this trend has not been examined for breast surgery.^1^

Our research aims to bridge this critical gap in knowledge. Given the rising prevalence of breast disease and the potential impact of reimbursement rates, this study aims to examine trends in Medicare reimbursements for common breast surgery operations. We hypothesize that reimbursement rates for common breast surgery operations have not kept pace with inflation. Examining these trends will illuminate the current financial landscape for breast surgery within Medicare, and understanding this landscape is crucial to ensuring access to high-quality, equitable breast cancer care.

## Methods

### Data Source

The Physician Fee Schedule Look-Up Tool, provided by the Centers for Medicare and Medicaid Services (CMS), serves as a comprehensive resource for deciphering Medicare reimbursements for physician services. It provides access to critical details for over 10,000 services encompassed within the Medicare Physician Fee Schedule (MPFS).^14^ Each operation is identified in the Physician Fee Schedule Look-Up Tool by their unique Current Procedural Terminology (CPT) code. These codes are maintained by the American Medical Association (AMA) CPT Editorial Panel. Since 1977, the AMA has continued a system for periodically updating the codes, which involves annually making additions, deletions, or revisions of existing CPT codes.^15^

### Data Acquisition

CPT codes for 10 common breast surgeries were identified by the authors to reflect the scope of a breast surgery practice (Table 1). Data for each code were obtained from the Physician Fee Schedule Look-Up Tool spanning from 2003 to 2023.^14^ During our study period, the CPT coding system underwent revisions for several breast cancer surgery procedures. These codes include 19301 (lumpectomy for malignancy), 19302 (lumpectomy with axillary dissection), 19303 (simple mastectomy), 19305 (radical mastectomy), and 19307 (modified radical mastectomy). To ensure the accuracy and consistency of our data across the entire study period, we identified the corresponding CPT codes used before the revisions. For example, in 2006, the CPT code for lumpectomy changed from 19160 to the current code, 19301. Therefore, we used code 19160 to identify lumpectomies performed before 2006. One code (CPT 38900, sentinel lymph node biopsy) was initially introduced in 2011 with no comparable prior equivalent; data collection and analysis for this operation was restricted to the period from 2011 to 2023.

**Table 1:** Breast surgery CPT codes evaluated

2003	2023	Operation name
*Breast surgery CPT codes*		
–	19100	Percutaneous core needle breast biopsy
–	19101	Open incisional breast biopsy
–	19120	Open excisional breast biopsy
19160	19301	Lumpectomy for malignancy
19162	19302	Lumpectomy w/axillary dissection
19180	19303	Simple mastectomy
19200	19305	Radical mastectomy
19240	19307	Modified radical mastectomy
–	38525	Biopsy/removal, lymph nodes
–	38900	Sentinel lymph node biopsy, injection^a^

*CPT* Current Procedural Terminology

^a^CPT code 38900 first appeared in 2011

**Fig. 1:**
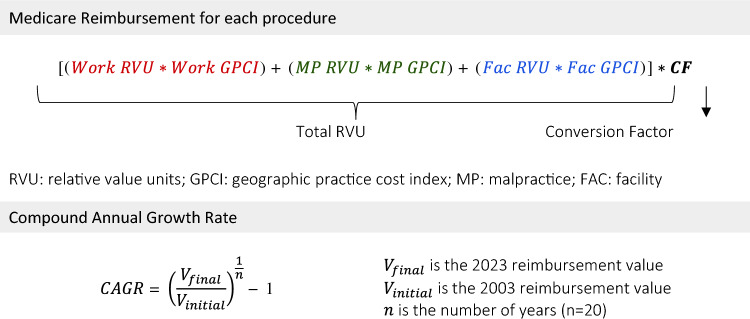
Reimbursement calculations. The top formula breaks down the components used to calculate Medicare reimbursement for each surgical procedure. This is calculated using the work, malpractice, facility RVUs, geographic adjustments, and the CF. The CF translates RVUs to a national dollar amount. The bottom formula calculates the average annual growth rate of Medicare reimbursement for each procedure over the study period (2003–2023). *CF* conversion factor

The core components of the Medicare compensation calculation include relative value units (RVUs), geographic practice cost indices (GPCIs), and the overall conversion factor (CF). RVUs assign a standardized value to each service based on its complexity (work involved), practice expenses, and malpractice liability. An RVU is further broken down into three subcomponents: work (wRVU), practice expense (peRVU), and malpractice (mpRVU). The total RVU for an operation is the sum of all three RVU types. Since practice costs vary geographically, the CMS employs GPCIs that assign a value multiplier to each Medicare payment locality, reflecting the relative cost of running a practice in that area. The GPCI is then applied to each component of a service’s RVU, ensuring reimbursement reflects location-specific expenses. The CF serves as a national dollar multiplier and converts geographically adjusted RVUs into a concrete dollar amount. This represents the authorized Medicare payment for a specific service in a particular location. This formula is shown in Fig. 1. The CMS conducts annual reviews to re-evaluate RVUs for each service and establish the national CF.

### Relative Value Unit (RVU) Trend Analysis

Changes in individual RVU components (wRVU, peRVU, and mpRVU) were assessed for each operation. Data on each RVU component for every operation were collected. The mean wRVU, peRVU, and mpRVU values for each operation were calculated annually, and the overall percentage change in wRVU, peRVU, and mpRVU from 2003 to 2023 was determined for each operation. The mean percentage change in each RVU category was then compared with the others (wRVU vs. peRVU vs. mpRVU).

### Reimbursement Trend Calculations

Medicare reimbursement for each operation was calculated at the locality level by multiplying the operation’s total RVU by the national CF. To arrive at a national annual reimbursement rate for each operation, these locality-specific reimbursements were then averaged across all Medicare payment localities (Fig. 1).

The yearly percentage change in Medicare reimbursement rates was calculated individually for each operation from 2003 to 2023. The average percentage change for all queried breast operations for 2003–2023 was then determined. To contextualize these changes, they were compared with the corresponding changes in the general consumer price index (CPI) over the same timeframe. The CPI, a well-established inflation metric in the US (measured in United States dollars [US$]), reflects the purchasing power of currency over time. By comparing reimbursement changes with CPI changes, we can assess how Medicare rates have fared against inflation rates. CPI data for each year (2003–2023) were obtained from the US Bureau of Labor Statistics and adjusted to reflect equivalent values in 2023 US$, ensuring consistent comparisons across the timeframe.^16^

To ensure a fair comparison of reimbursement rates across different years, all data from before 2023 were adjusted to reflect 2023 US$ values. This process, called inflation adjustment, eliminates the distorting effects of inflation, allowing us to see true changes in reimbursement over time. Once all values were converted into 2023 US$, a new, adjusted yearly percentage change in Medicare reimbursement was calculated individually for each operation. The percentage change for each operation over the time period was then compared. Focusing on percentage changes, rather than raw dollar amounts, provides a standardized way to compare reimbursement trends across operations and time periods.

To comprehensively analyze long-term growth trends in Medicare reimbursement rates, we employed the compound annual growth rate (CAGR) after converting all data into 2023 US$. CAGR surpasses the limitations of simply averaging annual changes by incorporating the concept of compounding. CAGR is a well-established financial metric for evaluating investment performance over time,^17^ and reflects the average annual growth rate an investment would achieve if all earnings were re-invested at the end of each year. By applying CAGR to inflation-adjusted reimbursement data for each operation, we gain a more nuanced understanding of long-term growth patterns. A positive CAGR value signifies an overall upward trajectory in reimbursement rates for a specific operation over the analyzed timeframe.

### Estimation of Real-Life Impact

To understand the potential financial impact of decreased Medicare reimbursements for breast cancer surgeries, we constructed a hypothetical scenario based on typical treatment patterns and the National Accreditation Program for Breast Centers (NAPBC) benchmark of 50% for breast-conserving surgery. We acquired 2023 national breast cancer case incidence data from ‘Cancer Facts and Figures’, published by the American Cancer Society.^1^ To estimate the number of breast cancer surgeries performed in a year, we made the following assumptions: 10% of patients present with Stage 4 disease (not requiring surgery) and 90% of patients undergo further surgical treatment. Of these, 50% of patients undergo breast-conservation surgery, 50% undergo mastectomy, and 80% undergo axillary surgery.

**Fig. 2:**
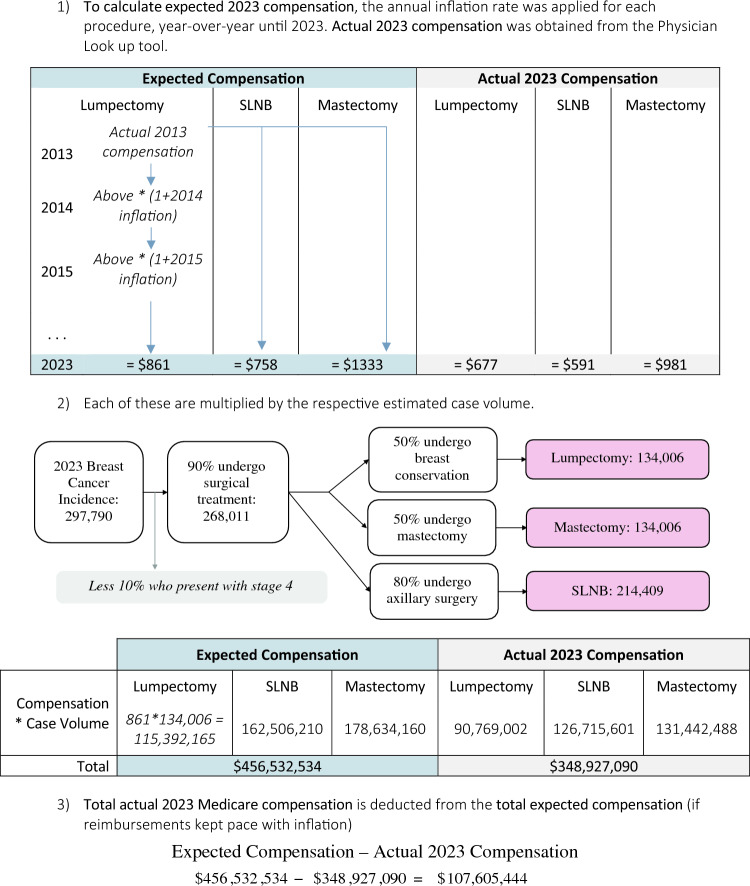
Sample calculation for the estimation of real-world impact. This figure illustrates the calculations for the potential financial impact of declining Medicare reimbursements for breast surgeries in 2023. Expected compensation represents what compensation would have been if 2013 reimbursement rates kept pace with inflation (hypothetical scenario). Actual 2023 compensation is obtained from the Physician Look-up tool (current reality). The difference is the estimated financial deficit due to declining reimbursements. *SLNB* sentinel lymph node biopsy

To estimate current total 2023 Medicare compensation, we obtained the current 2023 Medicare reimbursement rates for each relevant operation: lumpectomy for malignancy, sentinel lymph node biopsy, and simple mastectomy. These 2023 reimbursement rates were multiplied by the estimated case volumes for each operation. The products were then summed to arrive at the total Medicare compensation for breast cancer surgeries in 2023 under the current reimbursement scheme. To estimate the total compensation if Medicare reimbursements had kept pace with the rate of inflation, we obtained the 2013 Medicare reimbursement rates for each relevant operation (lumpectomy, lymph node biopsy, mastectomy). The CPI was then used to calculate the yearly inflation rate from 2013 to 2023. This inflation rate was then applied to ‘grow’ the 2013 reimbursement rates for each operation year-over-year until 2023. This represents the hypothetical scenario where reimbursements kept pace with inflation. Finally, we subtracted the actual total Medicare compensation for 2023 (calculated earlier) from the expected total compensation (if reimbursements kept pace with inflation). This difference represents the estimated financial impact of decreased reimbursement rates on breast surgeons in 2023. This workflow is shown in Fig. 2.

### Statistical Analysis

Mean scores were compared using a two-tailed t-test comparison of means or one-way analysis of variance (ANOVA). A *p* value < 0.05 was considered statistically significant. All statistical analysis was performed using SPSS version 29.0 (IBM Corporation, Armonk, NY, USA). This study qualified for Institutional Review Board exemption at our institution as it solely involved the analysis of publicly available online information and did not include any interaction with human subjects.

## Results

### RVU Analysis

As shown in Table 2, we found an increase in the averages for all RVU components (wRVU, peRVU, and mpRVU) from 2003 to 2023. Malpractice RVU had the greatest increase (+ 200.98 ± 91.08%), followed by peRVU (+ 37.40 ± 24.90%) and then wRVU (+ 18.21 ± 27.55%; *p* < 0.001). CPT codes 19303 (simple mastectomy) and 19301 (lumpectomy for malignancy) had the greatest increase in wRVU (70.45% and 69.12%, respectively), as well as in mpRVU (317.05% and 306.56%, respectively). CPT codes 19303 (simple mastectomy) and 19120 (excisional breast biopsy) saw the greatest increases in peRVU (70.32% and 67.64%, respectively). Conversely, CPT code 38900 (sentinel lymph node biopsy) saw no increases in wRVU, had the only decrease in peRVU (− 2.97%), and the smallest increase in mpRVU (9.43%).

**Table 2:** Changes in individual RVU components

	2003 RVU value	2023 RVU value	% change in RVU value	*p* value
*Work RVU [mean (SD)]*				
Overall	7.84 (5.41)	9.39 (6.37)	+ 18.21 (27.55)	<0.001^a^
Percutaneous core needle breast biopsy	1.27	1.27	0	<0.001^b^
Open incisional breast biopsy	3.18	3.23	+ 1.57	
Open excisional breast biopsy	5.56	5.92	+ 6.47	
Lumpectomy for malignancy	5.99	10.13	+ 69.12	
Lumpectomy w/axillary dissection	13.53	13.99	+ 3.4	
Simple mastectomy	8.80	14.00	+ 70.45	
Radical mastectomy	15.49	17.46	+ 12.72	
Modified radical mastectomy	16.00	17.99	+ 12.44	
Biopsy/removal, lymph nodes	6.07	6.43	+ 5.93	
Sentinel lymph node biopsy, injection^a^	2.50	2.50	0	
*Facility RVU [mean (SD)]*				
Overall	4.70 (3.15)	6.70 (4.53)	+ 37.40 (24.90)	<0.001
Percutaneous core needle breast biopsy	0.44	0.48	+ 9.09	<0.001
Open incisional breast biopsy	1.89	2.71	+ 43.49	
Open excisional breast biopsy	3.09	5.18	+ 67.64	
Lumpectomy for malignancy	4.52	7.18	+ 58.85	
Lumpectomy w/axillary dissection	7.88	9.74	+ 23.60	
Simple mastectomy	5.93	10.10	+ 70.32	
Radical mastectomy	9.07	12.47	+ 37.49	
Modified radical mastectomy	8.74	12.93	+ 47.94	
Biopsy/removal, lymph nodes	4.40	5.22	+ 18.64	
Sentinel lymph node biopsy, injection^a^	1.01	0.98	− 2.97	
*Malpractice RVU [mean (SD)]*				
Overall	0.79 (0.54)	2.30 (1.57)	+ 200.98 (91.08)	<0.001
Percutaneous core needle breast biopsy	0.10	0.30	+ 200.00	<0.001
Open incisional breast biopsy	0.20	0.30	+ 285.00	
Open excisional breast biopsy	0.56	1.44	+ 157.14	
Lumpectomy for malignancy	0.61	2.48	+ 306.56	
Lumpectomy w/axillary dissection	1.38	3.44	+ 149.28	
Simple mastectomy	0.88	3.67	+ 317.05	
Radical mastectomy	1.51	4.30	+ 184.77	
Modified radical mastectomy	1.62	4.43	+ 173.46	
Biopsy/removal, lymph nodes	0.48	1.57	+ 227.08	
Sentinel lymph node biopsy, injection^a^	0.53	0.58	+ 9.43	

*RVU* relative value unit, *SD* standard deviation

^a^Percentage change in each RVU category was compared with other RVU categories (work vs. facility vs. malpractice)

^b^Change for each operation within each RVU category was compared (work RVU core needle breast biopsy vs. work RVU open incisional breast biopsy vs. work RVU open excisional breast biopsy)

### Unadjusted Medicare Reimbursement Trends

From 2003 to 2023, the average change in Medicare reimbursement for all 10 breast operations was + 25.17 ± 24.90% (Table 3). During this same time, the CPI increased by + 69.15%, significantly more than the increase in reimbursement seen for all breast operations (*p* < 0.001). Of note, CPT codes 19303 (simple mastectomy) and 19301 (lumpectomy for malignancy) had the greatest increase in reimbursement (+ 71.30% and + 65.99%, respectively).

**Table 3:** Reimbursements, unadjusted and inflation-adjusted Medicare reimbursement changes for common breast operations from 2003 to 2023

		2003 average reimbursement (US$)	2023 average reimbursement (US$)	Unadjusted change in reimbursement (%)	Inflation-adjusted change in reimbursement (%)	Adjusted CAGR (%)
*All operations*						
All [mean (SD)]		526 (328)^a^	630.10 (427.13)	+ 25.17 (24.90)	− 20.70 (12.57)	− 1.45 (0.91)
*Individual operations*						
CPT code	Operation name					
19100	Percutaneous core needle breast biopsy	66	70	+ 5.16	− 36.41	− 2.24
19101	Open incisional breast biopsy	193	230	+ 19.05	−28.01	− 1.63
19120	Open excisional breast biopsy	338	430	+ 27.52	−22.89	− 1.29
19301	Lumpectomy for malignancy	408	677	+ 65.99	+ 0.37	+ 0.02
19302	Lumpectomy w/axillary dissection	835	930	+ 11.28	− 23.76	− 1.96
19303	Simple mastectomy	573	981	+ 71.30	+ 3.58	+ 0.18
19305	Radical mastectomy	956	1181	+ 23.57	− 25.28	− 1.45
19307	Modified radical mastectomy	966	1210	+ 25.25	− 24.27	− 1.38
38525	Biopsy/removal, lymph nodes	402	453	+ 2.42	− 24.30	− 2.29
38900	Sentinel lymph node biopsy, injection^b^	138	138	+ 0.14	− 25.99	− 2.48

All reimbursement values for 2003 and 2023 are rounded to the nearest whole US$ value

*SD* standard deviation, *CAGR* compound annual growth rate, *US$* United States dollar

^a^Excludes CPT 38900

^b^Values analyzed from 2011 to 2023

### Adjusted Medicare Reimbursement

After inflation adjustment for each operation to convert all dollar values to 2023 US$, we found that the mean inflation-adjusted percentage change for all studied operations over the study period was − 20.70 ± 12.57% (Table 3). CPT codes 19303 (simple mastectomy) and 19301 (lumpectomy for malignancy) were the only two operations with an increase in adjusted reimbursement (+ 3.58% and + 0.37%, respectively). For the remainder of the operations (all of which had a decrease in the adjusted reimbursement), CPT code 19100 (percutaneous core needle breast biopsy) experienced the greatest decrease (− 36.41%).

The adjusted average yearly CAGR for 2003–2023 was − 1.45 ± 0.91% for all studied operations. CAGR values followed a similar trend as adjusted Medicare reimbursement, with CPT codes 19303 (simple mastectomy) and 19301 (lumpectomy for malignancy) being the only queried codes to demonstrate a year-to-year growth in reimbursement (+ 0.18% annually and + 0.02% annually, respectively). Conversely, CPT codes 38900 (sentinel lymph node biopsy, injection), 38525 (biopsy/removal, lymph nodes), and 19100 (percutaneous core needle breast biopsy) had the greatest year-to-year decline in Medicare reimbursement (CAGR − 2.48%, − 2.29%, and − 2.24%, respectively).

### Estimation of Real-Life Impact

The 2023 estimated breast cancer case incidence was 297,790.^1^ After application of our assumptions about case composition, the following case volume estimations were obtained: 134,006 patients would undergo lumpectomy for malignancy in 2023, 134,006 patients would undergo simple mastectomy, and 214,409 patients would undergo sentinel lymph node biopsies (Fig. 2). The expected total 2023 compensation if reimbursements had grown at the rate of inflation was $456,532,534. The actual total 2023 compensation was $348,927,090. Thus, we estimate that breast surgeons were reimbursed $107,605,444 less for these surgeries in 2023 than if rates had kept pace with inflation over the past 10 years.

## Discussion

Our analysis reveals a concerning discrepancy between rising inflation and the current Medicare reimbursement for breast cancer surgery, with a 21% decrease in inflation-adjusted Medicare compensation since 2003. This misalignment has significant real-world consequences. Under the current system, breast surgeons stood to be reimbursed nearly $110 million less for these surgeries in 2023 compared with if rates had kept pace with inflation over the past decade. This decline threatens the financial sustainability of breast cancer surgery practices and has the potential to jeopardize access to essential care for Medicare beneficiaries.

Our findings add to a growing body of evidence highlighting declines in reimbursement rates across various surgical specialties, which report significant decreases in reimbursement rates after adjusting for inflation. Studies in colorectal surgery, vascular surgery, and abdominal transplantation report significant reimbursement decreases ranging from 11 to 23.2% over the past two decades.^5,8,18^ Similar trends hold true for general surgery, with inflation-adjusted declines exceeding 19.8%.^4,19^ This pattern also extends to select breast operations, as evidenced by a 13% decrease in reimbursement rates for reconstruction procedures over the past decade.^20^ With regard to oncologic breast operations, past studies examining reimbursement trends often focused on a limited number within a wider analysis of various surgical specialties. In contrast, our study is the first to comprehensively analyze trends for a broad range of breast surgery operations using Medicare data. This comprehensive approach allows for a more nuanced understanding of how reimbursement patterns differ across various breast cancer operations. For instance, Haglin et al. demonstrated stagnant or declining inflation-adjusted reimbursement for specific breast operations (e.g., 15.1% decrease for CPT code 19125, open excisional biopsy).^4^ Conversely, our analysis reveals that while most operations experienced a similar trend, lumpectomy (CPT code 19301) and simple mastectomy (CPT code 19303), two of the most common breast cancer operations, were exceptions and experienced slight inflation-adjusted increases of 0.37% and 3.58%, respectively. Interestingly, Hydrick et al. also observed increased reimbursement for these same procedures, although of much greater magnitude, reporting an overall 9% increase, driven primarily by a 39.3% increase for lumpectomy.^21^ These discrepancies likely stem from methodological differences, such as the specific data sets analyzed or variations in the study timeframes. Regardless of these variations, our findings, alongside the documented decline in reimbursements for breast reconstruction procedures, paint a concerning picture: inflation-adjusted Medicare reimbursements for most breast cancer care services are declining. This necessitates further investigation into the potential consequences for future access and quality of breast cancer care delivery for Medicare beneficiaries.

Declining inflation-adjusted Medicare reimbursements for breast cancer surgery operations pose significant challenges, with wide-ranging implications for patients and providers alike. First, this trend jeopardizes access to high-quality care for a substantial portion of the breast cancer population. Medicare plays a pivotal role in financing treatment for a large subset of patients.^3,22,23^ Lower reimbursements can have unintended consequences, potentially driving up overall costs for all patients, as a correlation between declining Medicare reimbursements and rising private insurance charges has previously been described.^13^ Additionally, hospitals, particularly safety-net hospitals serving predominantly minority patients or patients who already face systemic barriers to quality healthcare, may face greater financial strain due to declining reimbursements.^24^ This strain can make it difficult to invest in essential resources or programs that improve care delivery, potentially exacerbating existing racial disparities in breast cancer outcomes.^25,26^ Second, declining reimbursements present significant challenges for providers. Surgeons facing stagnant or declining rates may be less inclined to participate in Medicare networks, leading to deficiencies in access to, or longer wait times for, essential surgeries. Reduced reimbursements can also undermine the financial viability of physicians and medical institutions, restricting their capacity to deliver care or retain staff.^10^ Lastly, these reimbursement trends may disincentivize young surgeons from specializing in breast cancer surgery, leading to a potential workforce shortage in the future.^27^ These factors collectively impede the quality and accessibility of essential services for Medicare patients, potentially contributing to poorer breast cancer outcomes observed in Medicare beneficiaries compared with privately insured patients.^22,28,29^

Addressing declining Medicare reimbursements for breast cancer surgery is imperative to ensure continued access to high-quality, equitable breast cancer care. Effective action necessitates a comprehensive approach, starting with engagement in the Medicare RVU review process. RVUs are pivotal in determining procedure compensation, with regular reviews for every code mandated by the Omnibus Budget Reconciliation Act of 1990 to occur every 5 years.^30,31^ Specialty groups, such as the American Society of Breast Surgeons, can request off-cycle reviews for potentially undervalued codes, a process that involves gathering data from a minimum of 30 specialists per code regarding estimated time spent on various parts of a procedure, including pre-operative care, surgery, and post-operative care. Thus, surgeons can actively participate by identifying undervalued codes through practice data analysis, collaborating with professional societies to gather robust evidence for resource value scale update committee (RUC) review, and advocating for transparency in CMS code selection processes to ensure fair compensation for breast surgery operations. Additionally, surgeons can lobby for policy changes that address reimbursement rates and ensure fair compensation for breast cancer surgery operations.^31^ Prior research has shown that physician advocacy efforts can be effective in influencing healthcare policy and raising public awareness on key health economic issues.^32^ Finally, collaboration is key. Surgeons can collaborate with hospital administrators to advocate for fair reimbursement structures within the hospital system. Partnering with other healthcare providers, such as oncologists and radiologists, to create cost-effective care pathways can further improve overall value delivery.

This study offers a novel look at the collective decrease in Medicare reimbursement for breast surgery operations and provides perspective on the potential real-world impact of these changes. However, the analysis has limitations to consider. Focusing solely on Medicare reimbursements may overlook the potential impact on private insurers and the broader healthcare ecosystem. Additionally, while our estimate of case volume based on cancer incidence data provides a valuable starting point, it likely underestimates the true financial impact of decreased reimbursements on surgical breast care. For instance, our estimation does not consider benign excisions, nor additional surgery (such as re-excisions or completion axillary surgery that are required when there are unexpected findings on final pathology), therefore our estimates may in fact be lower than the actual reimbursement reductions experienced by breast surgeons. Furthermore, reimbursement rates are just one piece of the financial puzzle. Future research should explore provider overhead costs, staffing structures, and profit margins to offer a more comprehensive understanding of financial sustainability. Finally, our study does not examine how these trends affect patient outcomes. This is a critical area for further research, and future investigations should prioritize understanding the potential effects of this trend on patient access to specialists, provider treatment decisions, and long-term patient outcomes.

## Conclusion

Inflation-adjusted Medicare reimbursement rates for breast surgeries have declined from 2003 to 2023, and the estimated real-world deficit is immense. This downward trend, if left unaddressed, carries profound implications. Diminishing reimbursement rates may strain resources, potentially leading to staffing shortages and compromises in care quality. Surgeons, healthcare administrators, and policymakers must confront these impending challenges with proactive measures to mitigate these issues. Understanding reimbursement trends and their impacts provides the foundation for advocating for equitable policies and solutions, which are essential to ensure the accessibility and quality of breast surgery in the future.
